# Apigenin Attenuates Hepatic Ischemia–Reperfusion-Induced Lung Injury via Downregulation of MMP-3 and MCP-1: An Experimental Study in Rats

**DOI:** 10.3390/jcm14103530

**Published:** 2025-05-18

**Authors:** Chrysovalantis Mariorakis, Maria Lambropoulou, Panagoula Oikonomou, Christos Tsalikidis, Michail Pitiakoudis, Elissavet Anestiadou, Orestis Ioannidis, Alexandra K. Tsaroucha

**Affiliations:** 1 Postgraduate Program in Hepatobiliary/Pancreatic Surgery, Faculty of Medicine, Democritus University of Thrace, 68100 Alexandroupolis, Greece; cmari@auth.gr (C.M.); mlambro@med.duth.gr (M.L.); paoikono@med.duth.gr (P.O.); mpitiak@med.duth.gr (M.P.); atsarouc@med.duth.gr (A.K.T.); 2 Laboratory of Histology-Embryology, Faculty of Medicine, Democritus University of Thrace, 68100 Alexandroupolis, Greece; 3 Laboratory of Experimental Surgery, Faculty of Medicine, Democritus University of Thrace, 68100 Alexandroupolis, Greece; 4 Second Department of Surgery, Faculty of Medicine, Democritus University of Thrace, 68100 Alexandroupolis, Greece; 5 Fourth Department of Surgery, Medical School, Faculty of Health Sciences, Aristotle University of Thessaloniki, General Hospital “George Papanikolaou”, 57010 Thessaloniki, Greece; elissavetxatz@gmail.com; 6 Laboratory of Bioethics, Faculty of Medicine, Democritus University of Thrace, 68100 Alexandroupolis, Greece

**Keywords:** apigenin, immunohistochemistry, liver, lungs, ischemia–reperfusion injury, rats, experimental surgery

## Abstract

**Background/Objectives**: In liver transplant surgery, ischemia–reperfusion (I-R) maneuvers are frequently employed to control bleeding; however, such interventions can result in injury not only to the liver but also to remote organs. The lungs, in particular, are highly susceptible due to their extensive vascularization and inflammatory response. While pulmonary injury secondary to hepatic I-R is recognized, and despite the fact that various antioxidant compounds have been investigated for their potential to mitigate I-R-induced damage to hepatic tissue, few studies have focused on evaluating therapeutic agents aimed at mitigating lung damage in this setting. This study aimed to evaluate the protective effect of apigenin on pulmonary tissue following liver I-R injury using an experimental rat model. **Methods**: Sixty-three male albino Wistar rats (approximately 15 weeks old, weighing 220–350 g) were randomly allocated into three groups: a sham group (open–close surgery; *n* = 7), a control (C) group subjected to liver I-R injury only (*n* = 28), and an apigenin (Ap) group receiving intraperitoneal apigenin administration immediately after liver ischemia and prior to reperfusion (*n* = 28). Both the C and Ap groups were subdivided into four equal subgroups corresponding to euthanasia at 60-, 120-, 180-, and 240 min post-reperfusion. Lung tissues were harvested for immunohistochemical analysis targeting the expression of matrix metalloproteinase-3 (MMP-3) and monocyte chemoattractant protein-1 (MCP-1). **Results**: The apigenin-treated groups exhibited significantly reduced expression levels of MMP-3 and MCP-1 across all time points when compared to the control groups. In contrast, no expression of MMP-3 or MCP-1 was observed in the sham group. **Conclusions**: The findings support the protective role of the antioxidant apigenin in reducing pulmonary injury following liver I-R. The diminished expression of MMP-3 and MCP-1 in the apigenin-treated rats provides compelling evidence for its protective effects on remote organs.

## 1. Introduction

Liver tumor resections, liver transplantation, and vascular reconstructions are examples of surgical procedures that necessitate periods of ischemia followed by reperfusion. A well-known technique employed to induce temporary hepatic ischemia is the Pringle maneuver, which involves clamping the hepatic pedicle to obstruct blood flow through the hepatic vessels [1]. However, ischemia–reperfusion (I-R) is associated with tissue damage, known as I-R injury (IRI), which affects not only the liver but can also result in remote organ injury, most notably acute lung injury (ALI) [2,3]. Several studies have documented the systemic effects of hepatic IRI, including injury to distant organs such as the lungs, kidneys, and the intestine [4,5].

The pathogenesis of IRI-induced ALI is multifactorial, with cytokine-mediated inflammatory responses playing a central role [2,3,6]. Tumor necrosis factor-alpha (TNF-α) is a prototypical cytokine that significantly contributes to extracellular matrix degradation and tissue injury [7]. TNF-α also upregulates adhesion molecules, such as intercellular adhesion molecule-1 (ICAM-1) and E-selectin, promoting neutrophil recruitment to the pulmonary parenchyma [4]. Activated neutrophils exacerbate lung injury through the development of pulmonary edema and alveolar hemorrhage. In addition, oxidative stress—via the generation of reactive oxygen species (ROS)—intensifies tissue damage and may contribute to carcinogenesis [8,9].

To better understand the extent of hepatic IRI and its remote pulmonary effects, we focused on two biomarkers: monocyte chemoattractant protein-1 (MCP-1) and matrix metalloproteinase-3 (MMP-3). MCP-1 (also known as CCL2), a member of the CC chemokine family, is pivotal in initiating and sustaining inflammatory responses [10,11]. MMP-3, an extracellular matrix metalloproteinase, contributes to the remodeling of extracellular matrix components such as glycosaminoglycans, proteoglycans, elastin, laminin, and fibronectin [12,13,14,15]. Beyond its role in tissue regeneration and angiogenesis, MMP-3 is also implicated in neoplastic processes and inflammatory diseases such as arthritis [16,17].

Despite the known involvement of the lungs in hepatic IRI, there is a lack of studies investigating pharmacological interventions specifically targeting pulmonary protection in this context. Apigenin, a naturally occurring flavonoid found in various plants, has attracted attention due to its anti-inflammatory, antioxidant, antiviral, antimutagenic, antigenotoxic, and chemoprotective properties [18]. While it has been studied in various chronic diseases and malignancies, its potential role in mitigating remote organ injury following hepatic ischemia–reperfusion, particularly in the lungs, remains underexplored [19]. Existing literature on apigenin primarily addresses its role in hepatic or renal protection, with limited data concerning its effect on pulmonary inflammation and tissue remodeling following hepatic I-R [20,21]. Moreover, to the best of our knowledge, no previous experimental study has evaluated the expression of MMP-3 and MCP-1 in lung tissue as indicators of oxidative and inflammatory injury in this context. This study, therefore, offers novel insight into the potential protective role of apigenin on the lungs during liver I-R injury.

The aim of this study was to assess the effects of intraperitoneal administration of apigenin on IRI-induced acute lung injury. Specifically, we evaluated the expression levels of MMP-3 and MCP-1 in lung tissue as biomarkers of the inflammatory and extracellular matrix remodeling processes associated with hepatic IRI-induced ALI.

## 2. Materials and Methods

### 2.1. Experimental Animals

Sixty-three healthy male Wistar rats were used in this study. All animals underwent routine health screening in compliance with the guidelines of the Association for Assessment and Accreditation of Laboratory Animal Care International (AAALAC International) and the Federation of European Laboratory Animal Science Associations (FELASA). The experiments were conducted at the Laboratory of Experimental Surgery, Democritus University of Thrace, while histological examinations and immunohistochemical analyses were performed at the Laboratory of Histology and Embryology, Democritus University of Thrace. All necessary institutional approvals and protocols for laboratory animal care were obtained and followed. The experiment was performed, and results are reported, in accordance with the ARRIVE (Animal Research: Reporting of In Vivo Experiments) guidelines 2.0. [22].

### 2.2. Experimental Protocol

The Wistar rats were randomly allocated into three groups: sham, control (C), and apigenin (Ap). The rats were approximately 15 weeks old, weighing between 220 g and 350 g.

- Sham group (*n* = 7): Underwent an open–close surgical procedure without vascular clamping.
- Control (C) group (*n* = 28): Underwent 45 min of hepatic ischemia.
- Apigenin (Ap) group (*n* = 28): Underwent 45 min of hepatic ischemia followed by intraperitoneal administration of 5 mg apigenin (dissolved in 0.3 mL of 0.9% NaCl and 0.2 mL dimethyl sulfoxide [DMSO]). This dosage was selected based on its previously demonstrated efficacy in similar models of I-R injury [23].

The control and apigenin groups were further subdivided into four subgroups, each consisting of seven rats, based on euthanasia time points: 60, 120, 180, and 240 min post-reperfusion (C60, C120, C180, C240 and Ap60, Ap120, Ap180, Ap240, respectively).

Rats were anesthetized using diethyl ether for induction and maintained under sevoflurane anesthesia at a concentration of 5% in oxygen throughout the surgical procedure. A midline abdominal incision was performed in all groups. In the control and apigenin groups, hepatic ischemia was induced using the Pringle maneuver by applying a microvascular clip to the hepatic pedicle for 45 min, followed by clip removal to allow reperfusion. The duration of hepatic ischemia was selected based on previous studies demonstrating that this duration reliably produces significant ischemia–reperfusion injury while maintaining a high survival rate in rat models [24]. This duration allows for the assessment of both early inflammatory responses and the protective effects of pharmacological interventions such as apigenin. Apigenin was administered intraperitoneally immediately before reperfusion in the apigenin group.

At the end of the experiment, euthanasia was performed by continuing sevoflurane administration at 5% for at least 5 min to ensure deep anesthesia, followed by cardiac puncture and exsanguination. All procedures complied with European Union Directive 2010/63/EU on animal experimentation. Following the assigned euthanasia time points, lung tissues were harvested and immediately fixed in formalin.

### 2.3. Immunohistochemistry

Lung tissue samples were carefully dissected, immediately fixed in 10% formaldehyde, embedded in paraffin wax, serially sectioned, and stained following standard histological protocols. The investigation was specifically designed to assess immunohistochemical expression of MMP-3 and MCP-1 in lung tissue, which served as the primary outcome measures reflecting inflammatory and extracellular matrix remodeling responses following hepatic ischemia–reperfusion injury. Unstained 4 μm histological sections from each paraffin block were treated with 0.3% H_2_O_2_ for 15 min at room temperature to block endogenous peroxidase activity. Immunostaining was performed using the Super Sensitive One-Step Polymer HRP Detection System (QD 630-XAKE, Biogenex, Fremont, CA, USA) according to the manufacturer’s instructions.

The sections were incubated for 60 min with the following primary antibodies:

- *MMP-3 (rabbit polyclonal, Novus Biologicals, Centennial, CO, USA; catalog no. NB100-91878), diluted 1:70.*
- *MCP-1 (mouse monoclonal, Merck Millipore, Burlington, MA, USA; catalog no. MABN712), uMABN712), diluted 1:200.*

Control sections were incubated with non-immunized rabbit serum. Antibody binding was visualized using 0.05% diaminobenzidine (DAB) chromogen for 10 min. Sections were counterstained with Mayer’s hematoxylin, mounted, and examined under a Nikon Eclipse 50i light microscope (Nikon Instruments Inc., Melville, NY, USA).

### 2.4. Evaluation of Antibody Expression

Antibody expression was quantified by counting the number of stained cells. The labeling index was graded as follows:

- Negative (0): <10% of cells stained.
- Low (1): 10–30% of cells stained.
- Moderate (2): 30–70% of cells stained.
- High (3): >70% of cells stained.

### 2.5. Statistical Analysis

Statistical analysis of the immunohistochemistry results was performed using the R programming language and SPSS software version 25.0 (SPSS Inc., Chicago, IL, USA). Although P-values were not calculated, statistical comparisons were conducted using Bayes factor analysis, which is suitable for categorical data with small sample sizes. Interpretation followed the classification by Kass and Raftery [25], providing graded levels of evidence rather than binary significance thresholds.

The Bayes factor (BF) test was applied using the R Bayes Factor package to assess equality and inequality hypotheses. The Bayes factor (BFH1/H2) quantifies the likelihood of one hypothesis (H1) over an alternative (H2). For example, if BFH1/H2 = 3, the data suggest that H1 is three times more likely than H2.

According to Kass and Raftery’s interpretation of Bayes factor values:

- BF = 1–3: Weak evidence.
- BF = 3–20: Positive evidence supporting H1.
- BF = 20–150: Strong evidence.
- BF > 150: Very strong evidence.

## 3. Results

### 3.1. Immunohistochemical Analysis

Immunohistochemical staining was used to evaluate the expression of matrix metalloproteinase-3 (MMP-3) and monocyte chemoattractant protein-1 (MCP-1) in lung tissue across different time points following hepatic ischemia–reperfusion (I-R) injury. Comparisons were made between the control (C) and apigenin-treated (Ap) groups, as well as with the sham group.

#### 3.1.1. MMP-3 Expression

In the sham group, all animals exhibited negative MMP-3 staining across all time points. In the C60 vs. Ap60 comparison, weak evidence of a difference was observed (Bayes factor [BF] = 3.0). Stronger differences emerged at later time points:

C120 vs. Ap120: BF = 30
C180 vs. Ap180: BF = 43
C240 vs. Ap240: BF = 85

These findings indicate a progressive and statistically supported reduction in MMP-3 expression in the apigenin-treated groups compared to controls over time.

**Figure 1:**
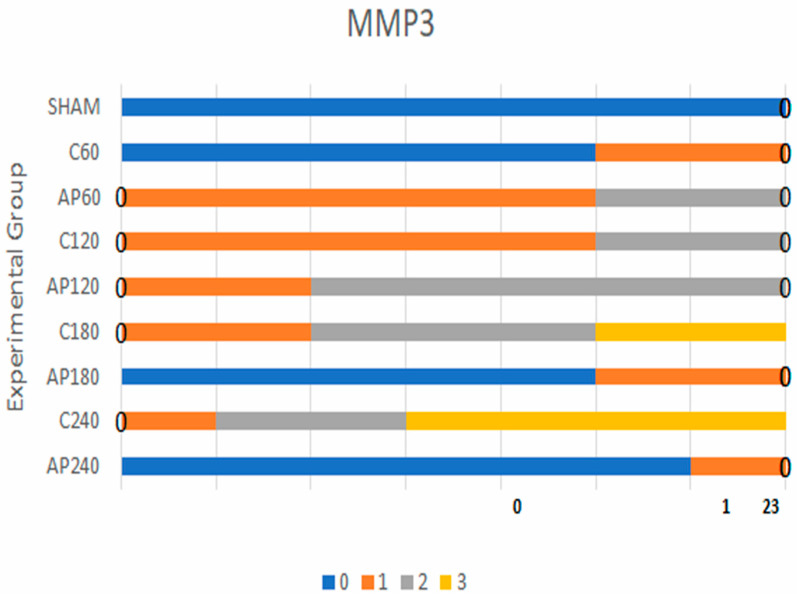
Mosaic plot depicting MMP-3 expression levels. Blue represents the lowest expression, orange the second lowest, gray the second highest, and yellow the highest.

**Figure 2:**
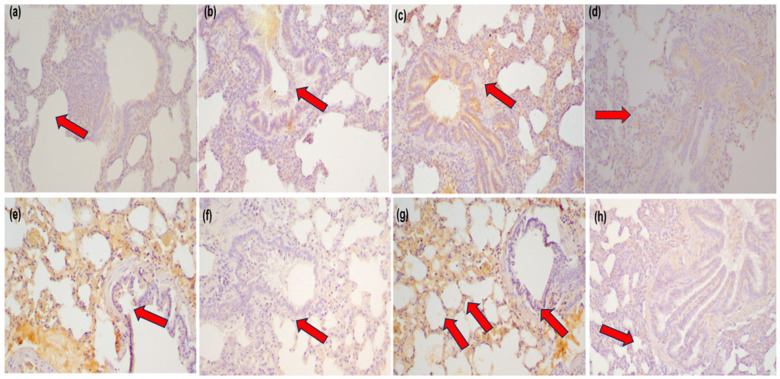
Photomicrographs of MMP-3 expression in lung tissue of the control and apigenin groups at 120 min (**a**,**b**), 180 min (**c**,**d**), and 240 min (**e**,**f**) and MCP-1 expression at 180 min (**g**,**h**) (magnification ×200). Arrows indicate areas of positive immunohistochemical staining.

Based on the results for MMP-3 expression (Figure 1), weak statistical significance was observed in the comparisons between sham vs. C60 and C60 vs. Ap60. In contrast, the comparisons between C120 vs. Ap120 (Figure 2a,b), C180 vs. Ap180 (Figure 2c,d), and C240 vs. Ap240 (Figure 2e,f) demonstrated strong statistical significance, indicating a pronounced reduction in MMP-3 expression following apigenin treatment.

#### 3.1.2. MCP-1 Expression

MCP-1 expression followed a similar pattern. Sham group animals consistently showed negative staining. Weak evidence for a difference was found in early time point comparisons (sham vs. C60, C60 vs. Ap60, C120 vs. Ap120), with BF values of 1.7, 3.2, and 9.0, respectively.

In contrast, later comparisons yielded much stronger statistical evidence:

C180 vs. Ap180: BF = 470 (very strong)
C240 vs. Ap240: BF = 90 (strong)

**Figure 3:**
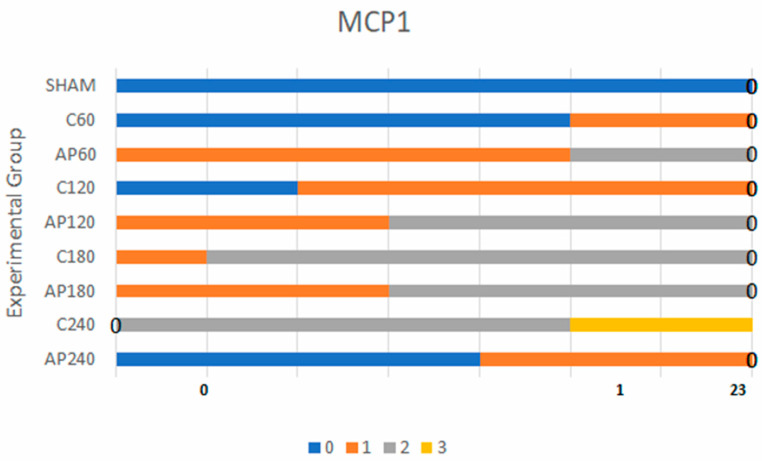
Mosaic plot depicting MCP-1 expression levels. Blue represents the lowest expression, orange the second lowest, gray the second highest, and yellow the highest.

These results support a time-dependent anti-inflammatory effect of apigenin, with substantial reductions in MCP-1 expression at 180 and 240 min post-reperfusion (Figure 3). More specifically, weak statistical significance was found in the comparisons of sham vs. C60, C60 vs. Ap60, and C120 vs. Ap120. However, the comparisons between C180 vs. Ap180 (Figure 2g,h) and C240 vs. Ap240 revealed very strong and strong statistical significance, respectively, further supporting the anti-inflammatory effect of apigenin over time.

MMP-3 and MCP-1 expression levels in the sham group were minimal and not statistically significant when compared to the control group. For MMP-3, the highest statistically significant differences were found in the comparisons between C120 vs. Ap120, C180 vs. Ap180, and C240 vs. Ap240. Similarly, for MCP-1, the comparison between C180 and Ap180 yielded the strongest statistical significance. All findings were derived using Bayes factor analysis (Table 1 and Table 2) [25].

## 4. Discussion

The suppression of MMP-3 and MCP-1 by apigenin observed in this study likely reflects its ability to modulate key inflammatory and remodeling pathways.

This experimental study demonstrated that apigenin may exert a protective effect against acute lung injury associated with hepatic ischemia–reperfusion (IRI) in male rats. The protective action was evidenced by the reduced expression levels of MMP-3 and MCP-1 in the apigenin-treated groups compared to controls. These findings suggest that apigenin may attenuate the inflammatory response and tissue remodeling processes in lung tissue secondary to hepatic IRI. This is consistent with the known pathophysiology of hepatic IRI, where oxidative stress and the release of inflammatory mediators such as TNF-α, IL-6, and ICAM-1 lead to systemic endothelial dysfunction and infiltration of neutrophils in remote organs, including the lungs. Activation of Kupffer cells, increased ROS generation, and endothelial swelling further exacerbate tissue damage, contributing to pulmonary edema and hemorrhage [26,27,28,29]. In addition, apigenin has been shown to inhibit NF-κB activation, which plays a central role in the transcription of genes encoding cytokines and matrix-degrading enzymes [30]. By inhibiting this pathway, apigenin may reduce leukocyte recruitment and limit pulmonary matrix degradation, thereby attenuating the remote organ damage caused by hepatic IRI.

While our findings highlight notable differences in lung tissue markers between the control and apigenin-treated groups, it is essential to interpret these results as associative rather than causative. The study was designed to explore correlations between apigenin administration and inflammatory responses following hepatic ischemia–reperfusion injury. Due to the absence of randomization, mechanistic pathway analysis, or pharmacokinetic confirmation, a direct causal effect of apigenin on the observed outcomes cannot be firmly established. These results should be viewed as preliminary and hypothesis-generating, warranting further investigation through mechanistic studies and randomized controlled experiments to clarify the potential therapeutic role of apigenin.

The increased expression of MMP-3 and MCP-1 in the control group is consistent with the findings of Palladini et al. [31] and Moench et al. [32], who reported similar elevations in lung tissue following hepatic ischemia–reperfusion. More specifically, Palladini et al. [31] reported increased MMP-3 expression in the pulmonary parenchyma of male Sprague–Dawley rats following hepatic IRI, consistent with our control group findings. Similarly, Moench et al. [32] documented elevated MCP-1 expression in the lungs under hepatic IRI conditions. These pro-inflammatory mediators are well known to contribute to remote organ damage, particularly acute lung injury, through extracellular matrix degradation and immune cell recruitment. While these findings establish the role of MMP-3 and MCP-1 in IRI-induced pulmonary inflammation, our study is the first to demonstrate that apigenin significantly suppresses MCP-1 expression, highlighting its potential therapeutic role in mitigating remote pulmonary inflammation.

The pathophysiology of hepatic IRI-induced lung injury is complex and multifactorial. It involves a cascade of inflammatory cytokines (TNF-α, IL-6, IL-18), adhesion molecules (ICAM), neuropeptides such as substance P, platelet-activating factor (PAF), and cytokine-induced neutrophil chemoattractants [33]. Oxidative stress also plays a crucial role, as the excessive production of reactive oxygen species (ROS) promotes pulmonary edema and alveolar hemorrhage [5,34]. Through its antioxidant activity, apigenin could stabilize cellular membranes, reduce neutrophil recruitment, and preserve nitric oxide (NO) availability, which is typically depleted under oxidative stress, leading to vasoconstriction and impaired microcirculation [35,36]. In our study, we observed that hepatic IRI led to significant upregulation of MCP-1 and MMP-3 in the lung tissue, both of which play critical roles in promoting inflammatory cell recruitment and extracellular matrix remodeling. Notably, treatment with apigenin resulted in a marked reduction in the expression of both mediators, suggesting a suppressive effect on the pulmonary inflammatory response triggered by hepatic IRI. Although MCP-1 was the only inflammatory marker directly measured, the downregulation of this key chemokine, along with MMP-3, supports the anti-inflammatory potential of apigenin. These findings are consistent with previous reports that apigenin modulates inflammation through inhibition of the NF-κB pathway and exerts antioxidant effects by scavenging reactive oxygen species and enhancing the expression of endogenous antioxidant enzymes such as superoxide dismutase and glutathione peroxidase [37,38,39]. Therefore, our data suggest that apigenin may attenuate remote organ injury not only by reducing chemokine-mediated inflammation but also potentially through its broader antioxidant and anti-apoptotic effects [37,38,40]. Previous studies investigating antioxidant therapies in hepatic IRI have primarily focused on hepatic protection, with limited evaluation of pulmonary consequences. While some agents (e.g., N-acetylcysteine, melatonin) have shown protective effects on lung tissue in similar models, there is a paucity of evidence regarding flavonoid-based compounds [41].

Several experimental interventions have been explored to protect lung tissue from IRI-related damage. Rahman et al. [42] demonstrated that CD40L administration reduced MMP-mediated pulmonary injury. Kollara et al. [37] showed that silibinin, an antioxidant administered before reperfusion, decreased MMP-2, MMP-3, and MMP-9 expression in the lung and kidney. Similarly, Zhou et al. [43] reported that sufentanil attenuated hepatic inflammation by limiting MCP-1-mediated phagocyte recruitment. Moench et al. [32] further confirmed that MCP-1 expression increases during liver transplantation involving ischemia–reperfusion maneuvers. In addition, studies by Tsaroucha et al. [23] and Tsalkidou et al. [38] demonstrated the hepatoprotective effects of apigenin, with Tsalkidou et al. highlighting its modulation of Fas/FasL gene expression as a possible mechanism of action. Additionally, previous research has shown that apigenin can regulate apoptosis-related genes, such as Fas and FasL, and enhance the expression of antioxidant enzymes, reinforcing its protective capacity in ischemia–reperfusion contexts [39]. Our findings add to a growing body of research suggesting that naturally derived polyphenols, such as apigenin, can offer multiorgan protection by modulating systemic oxidative and inflammatory cascades.

Our findings align with prior research demonstrating apigenin’s anti-inflammatory and cytoprotective effects in ischemia–reperfusion models. In hepatic IRI, apigenin has been shown to reduce ICAM-1 expression, increase BCL-2 levels, and decrease BAX expression, indicating anti-apoptotic and endothelial-protective activity [23]. In testicular torsion–detorsion models, apigenin significantly reduced apoptotic cell counts and preserved tissue structure [40]. In pancreatitis-induced lung injury, apigenin was associated with decreased TNF-α, IL-6, and MPO expression, indicating attenuation of both cytokine-mediated and oxidative damage [44]. Mechanistically, these effects have been linked to inhibition of NF-κB signaling and suppression of proinflammatory gene expression, including TNF-α and COX-2 [45,46].

MCP-1 is a chemokine that promotes monocyte and macrophage recruitment to injured tissues, contributing to the amplification of the inflammatory cascade in hepatic and remote organ IRI [23]. Its upregulation has been linked to systemic inflammatory cascades following liver IRI, contributing to leukocyte infiltration, monocyte/macrophage infiltration, endothelial dysfunction, and remote organ damage, including acute lung injury [47]. In the context of hepatic IRI, increased MCP-1 expression in remote organs such as the lungs has been linked to acute lung injury (ALI) via NF-κB activation and endothelial dysfunction [48]. The observed reduction in MCP-1 expression in the apigenin-treated group suggests a possible downregulation of monocyte-mediated inflammation in the lungs.

MMP-3, a matrix metalloproteinase, facilitates extracellular matrix degradation, promotes neutrophil infiltration, and plays a role in tissue remodeling during inflammation [49]. Elevated MMP-3 activity has been associated with tissue damage and remodeling in various models of inflammatory lung injury. In our study, the upregulation of both markers in the control group and their significant suppression in the apigenin-treated group suggest that apigenin exerts its protective effect at least in part by downregulating these mediators. This may be achieved through inhibition of NF-κB signaling, which regulates MCP-1 and MMP-3 expression, and through enhancement of the antioxidant defense system, thereby interrupting the feedback loop of oxidative stress and inflammation in remote lung tissue. In our study, intraperitoneal administration of apigenin effectively reduced pulmonary MMP-3 and MCP-1 expression, particularly at 120 and 240 min post-reperfusion. This time-dependent response suggests that apigenin may exert its protective effects by modulating the inflammatory cascade and limiting oxidative damage during the critical early phases of reperfusion. Apigenin was administered acutely to specifically evaluate its protective effects during the critical reperfusion phase, which is characterized by a surge of oxidative stress and cytokine release. Chronic administration models, while informative, may not isolate the acute-phase mechanisms relevant to surgical settings like transplantation or resection [50]. Although we did not measure plasma levels of apigenin in this study, previous pharmacokinetic studies in rats have shown that intraperitoneal administration of apigenin results in rapid systemic absorption, with peak plasma concentrations typically observed within 1–2 h [51]. These dynamics support its use in an acute setting to mitigate IRI-related injury.

From a translational standpoint, lung complications following hepatic surgery or transplantation represent a significant source of morbidity. The present findings, if confirmed in further studies, suggest that apigenin may serve as a candidate agent for mitigating such complications. Its availability, low toxicity profile, and broad anti-inflammatory action make it a promising adjunct in perioperative or post-transplant care. To our knowledge, this is the first study to evaluate the effects of apigenin on pulmonary MMP-3 and MCP-1 expression in a hepatic IRI model. The temporal analysis across four reperfusion time points adds further granularity to our understanding of the early remote inflammatory response and the therapeutic window for intervention.

This study presents certain limitations that should be acknowledged. First, the use of a small animal model may limit the generalizability of the findings to human physiology. Second, while the experimental design included standardized ischemia–reperfusion timing and histological evaluation, plasma concentrations of apigenin were not measured, which restricts conclusions about its pharmacokinetic profile and systemic bioavailability. Third, the study focused on two key biomarkers—MMP-3 and MCP-1—as indicators of inflammation and tissue remodeling. Although these are relevant and well-characterized mediators, they do not fully represent the complexity of inflammatory and oxidative pathways involved in acute lung injury. Additionally, the use of a single apigenin dose (5 mg/kg) precludes assessment of dose-dependent effects, and the short observation period (up to 240 min post-reperfusion) limits insight into long-term outcomes. Due to financial constraints, formal histopathological scoring was not performed, which limits the objectivity and quantitative interpretation of lung tissue damage. Another limitation of the present study is the lack of quantitative assessment of apoptosis in lung tissue. Although histological and immunohistochemical indicators of tissue injury were observed, apoptosis-specific assays, such as TUNEL staining or caspase-3 detection, were not included. Future studies should integrate these methods to better elucidate the cellular mechanisms underlying apigenin’s protective effects. Additionally, the study did not include measurements of oxidative stress markers such as malondialdehyde (MDA) or antioxidant enzyme levels, which would have provided further insight into the redox-modulating effects of apigenin. Finally, another limitation of the study is the absence of a separate solvent control group. Although the DMSO concentration used was minimal and is not expected to significantly affect inflammatory markers, the inclusion of a vehicle-only group would have allowed more definitive attribution of observed effects solely to apigenin.

Future research should aim to address the limitations of the present study by employing a more comprehensive experimental approach. This includes incorporating additional inflammatory and signaling markers, such as tumor necrosis factor-alpha (TNF-α), interleukin-6 (IL-6), and nuclear factor kappa B (NF-κB), to better elucidate the underlying mechanisms of lung injury and apigenin’s anti-inflammatory effects. Direct assessments of oxidative stress parameters should also be undertaken. Pharmacokinetic analyses and dose–response studies will be essential to optimize the route, timing, and concentration of apigenin administration. Moreover, the implementation of standardized histopathological grading systems will enhance the objectivity of tissue evaluation. Future studies should also include quantitative assessments of apoptosis, such as TUNEL or caspase-based assays, to better characterize the extent of cell death and the anti-apoptotic potential of apigenin in lung tissue following hepatic ischemia–reperfusion injury. Long-term outcome studies and validation in larger animal models—or eventual translation to clinical settings—will be crucial to evaluate the sustained efficacy and safety of apigenin in the context of hepatic ischemia–reperfusion-induced pulmonary injury.

## 5. Conclusions

This experimental study demonstrated that intraperitoneal administration of apigenin was associated with reduced expression of MMP-3 and MCP-1 in lung tissue following hepatic ischemia–reperfusion in rats. These findings suggest that apigenin may modulate inflammatory and extracellular matrix remodeling responses in the lungs, as indicated by the expression of these two markers. However, due to the absence of additional analyses—such as histopathological scoring, apoptosis detection, or antioxidant profiling—these results should be considered preliminary. Further research incorporating a broader set of outcome measures is needed to comprehensively evaluate the pulmonary protective potential of apigenin in this context.

## Figures and Tables

**Table 1:** Expression levels of MMP-3 and MCP-1 in lung tissue and corresponding Bayes factor values across experimental groups.

Parameter	Comparison	Bayes Factor	Level 0 (n)	Level 1 (n)	Level 2 (n)	Level 3 (n)
**MMP-3**	Sham vs. C60	1.8	Sham: 7, C60: 5	Sham: 0, C60: 2	Sham: 0, C60: 0	Sham: 0, C60: 0
	C60 vs. Ap60	3.0	C60: 5, Ap60: 5	C60: 2, Ap60: 2	C60: 0, Ap60: 0	C60: 0, Ap60: 0
	C120 vs. Ap120	30	C120: 0, Ap120: 0	C120: 5, Ap120: 3	C120: 2, Ap120: 4	C120: 0, Ap120: 0
	C180 vs. Ap180	43	C180: 0, Ap180: 0	C180: 2, Ap180: 3	C180: 3, Ap180: 4	C180: 2, Ap180: 0
	C240 vs. Ap240	85	C240: 0, Ap240: 1	C240: 2, Ap240: 6	C240: 4, Ap240: 0	C240: 0, Ap240: 0
**MCP-1**	Sham vs. C60	1.7	Sham: 7, C60: 5	Sham: 0, C60: 2	Sham: 0, C60: 0	Sham: 0, C60: 0
	C60 vs. Ap60	3.2	C60: 5, Ap60: 5	C60: 2, Ap60: 2	C60: 0, Ap60: 0	C60: 0, Ap60: 0
	C120 vs. Ap120	9.0	C120: 0, Ap120: 0	C120: 5, Ap120: 3	C120: 0, Ap120: 4	C120: 0, Ap120: 0
	C180 vs. Ap180	470	C180: 0, Ap180: 0	C180: 1, Ap180: 0	C180: 6, Ap180: 4	C180: 0, Ap180: 0
	C240 vs. Ap240	90	C240: 0, Ap240: 0	C240: 1, Ap240: 0	C240: 2, Ap240: 3	C240: 4, Ap240: 0

Abbreviations: MMP-3, matrix metalloproteinase-3; MCP-1, monocyte chemoattractant protein-1; Ap, apigenin-treated group; C, control group; n, number of rats in each expression level. Note: Expression levels: Level 0 = negative expression (<10%), Level 1 = low expression (10–30%), Level 2 = moderate expression (30–70%), Level 3 = high expression (>70%).

**Table 2:** Summary of statistical comparisons using Bayes factor (BF) analysis for MMP-3 and MCP-1 expression in lung tissues.

Marker	Group Comparison	Bayes Factor	Interpretation
**MMP-3**	C60 vs. Ap60	3.0	Weak evidence
**MMP-3**	C120 vs. Ap120	30	Strong evidence
**MMP-3**	C180 vs. Ap180	43	Strong evidence
**MMP-3**	C240 vs. Ap240	85	Strong evidence
**MCP-1**	C60 vs. Ap60	3.2	Weak evidence
**MCP-1**	C120 vs. Ap120	9.0	Positive evidence
**MCP-1**	C180 vs. Ap180	470	Very strong evidence
**MCP-1**	C240 vs. Ap240	90	Strong evidence

Bayes factor values were interpreted according to the classification proposed by Kass and Raftery, 1995 [25]: BF = 1–3 indicates weak evidence, BF = 3–20 positive evidence, BF = 20–150 strong evidence, and BF > 150 very strong evidence.

## Data Availability

Data are available upon request at cmari@auth.gr.

## Abbreviations

The following abbreviations are used in this manuscript:

Abbreviation	Full Term
ALI	Acute Lung Injury
Ap	Apigenin-treated group
ARRIVE	Animal Research: Reporting of In Vivo Experiments
BF	Bayes Factor
C	Control group
CD40L	CD40 Ligand
DAB	Diaminobenzidine
DMSO	Dimethyl Sulfoxide
ICAM-1	Intercellular Adhesion Molecule-1
INF-γ	Interferon-gamma
IL-1	Interleukin-1
IL-6	Interleukin-6
IL-18	Interleukin-18
I-R	Ischemia-Reperfusion
IRI	Ischemia-Reperfusion Injury
MCP-1	Monocyte Chemoattractant Protein-1
MMP-3	Matrix Metalloproteinase-3
NaCl	Sodium Chloride
NF-κB	Nuclear Factor kappa-light-chain-enhancer of activated B cells
NO	Nitric Oxide
ROS	Reactive Oxygen Species
Sham	Sham-operated group
TNF-α	Tumor Necrosis Factor-alpha
